# *In Vitro* Screening for **β**-Hydroxy-**β**-methylglutaryl-CoA Reductase Inhibitory and Antioxidant Activity of Sequentially Extracted Fractions of *Ficus palmata* Forsk

**DOI:** 10.1155/2014/762620

**Published:** 2014-05-06

**Authors:** Danish Iqbal, M. Salman Khan, Amir Khan, Mohd. Sajid Khan, Saheem Ahmad, Ashwani K. Srivastava, Paramdeep Bagga

**Affiliations:** ^1^Clinical Biochemistry and Natural Product Research Lab, Department of Biosciences, Integral University, Lucknow 226026, India; ^2^School of Life Sciences, Glocal University, Saharapur, Mirzapur 247121, India; ^3^Faculty of Pharmacy, Integral University, Lucknow 226026, India

## Abstract

Hypercholesterolemia-induced oxidative stress has been strongly implicated in the pathogenesis of atherosclerosis, which is one of the major causes of mortality worldwide. The current work, for the first time, accounts the antioxidant, genoprotective, antilipoperoxidative, and HMG-CoA reductase (EC 1.1.1.34) inhibitory properties of traditional medicinal plant, *Ficus palmata *Forsk. Our result showed that among sequentially extracted fractions of *Ficus palmata *Forsk, FPBA (*F. palmata* bark aqueous extract) and FPLM (*F. palmata* leaves methanolic extract) extracts have higher phenolic content and also exhibited significantly more radical scavenging (DPPH and Superoxide) and antioxidant (FRAP) capacity. Moreover, FPBA extract also exhibited significantly higher inhibition of lipid peroxidation assay. Additionally, results showed almost complete and partial protection of oxidatively damaged DNA by these plant extracts when compared to mannitol. Furthermore, our results showed that FPBA extract (IC_50_ = 9.1 ± 0.61 µg/mL) exhibited noteworthy inhibition of HMG-CoA reductase activity as compared to other extracts, which might suggest its role as cardioprotective agent. In conclusion, results showed that FPBA extract not only possess significant antioxidant and genoprotective property but also is able to attenuate the enzymatic activity of HMG-CoA reductase, which might suggest its role in combating various oxidative stress-related diseases, including atherosclerosis.

## 1. Introduction

Hypercholesterolemia and its induced oxidative stress are now considered to be one of the major contributors in progression of atherosclerosis [1]. An excessive concentration of lipids in plasma may alter the lipoprotein metabolism and results in low density lipoprotein (LDL) accumulation in subendothelial space of arteries where it undergoes oxidative modifications to form oxidized LDL [2], which is highly atherogenic [3]. Several risk factors like hypercholesterolemia and cholesterol-induced oxidative stress enhance the formation of reactive oxygen species (ROS) which leads to the advancement of atherosclerotic lesions in vascular wall [4, 5]. Studies have reported that elevated lipid level, like total cholesterol (TC), triglyceride (TG), and low density lipoprotein (LDL) cholesterol, and a decrease in high density lipoprotein (HDL) cholesterol are directly associated with hyperlipidemia and atherosclerosis [6]. The cholesterol synthesis is regulated by *β*-hydroxy-*β*-methylglutaryl-CoA reductase (HMG-CoA reductase) (EC 1.1.1.34), the rate limiting enzyme of cholesterol pathway [7], and catalyzes the conversion of HMG-CoA to mevalonic acid. Currently prescribed drugs that lower cholesterol level mainly work by inhibiting the HMG-CoA reductase enzymatic activity [8, 9]. Treatment and management of hyperlipidemia include dietary changes, weight loss, and use of hypolipidemic drugs. Nonetheless, these oral medications have certain limitation and side effects [10]. Therefore, naturally derived therapeutic agents are in high demand for the treatment of hyperlipidemia and cholesterol-induced oxidative stress as well as atherosclerosis. Plant products are less toxic, have no side effects and free radical scavengers, and are now considered to be the best source for new hypolipidemic drugs and atherosclerosis development programme [11–14]. The existence of cholesterol lowering agents and HMG-CoA reductase inhibitors has been demonstrated in different plant species including garlic [15],* Ananas comosus* [16], kiwifruit [17], and* Gynostemma pentaphyllum* [18].* Ficus palmata* Forsk (Moraceae), commonly known as wild fig, is used traditionally in the treatment of constipation and diseases of the lungs and bladder [19]. Leaves, bark, and heartwood of* F. palmata* contain *β*-sitosterol and a new tetracyclic triterpene-glaunol acetate. Besides, ceryl behenate, lupeol, and *α*-amyrin acetate are reported from the stem bark of* F. palmata *[20]. Since other* Ficus* species like* F. benghalensis* [21],* F. carica* [22], and* F. virens* [14] are known to possess antioxidant and other pharmacological properties, the present study was initially projected to illustrate the antioxidant, genoprotective, and antilipoperoxidative as well as HMG-CoA reductase inhibitory properties of various sequentially extracted fractions of* F. palmata*.

## 2. Material and Methods

All the chemicals used in this study were of analytical grade and procured from HiMedia Laboratories, Mumbai, India, and Merck India. The HMG-CoA reductase assay kit was procured from Sigma-Aldrich (St. Louis, MO, USA).

### 2.1. Plant Collection and Preparation of Extracts

The fresh plant materials, namely, leaves and stem bark of* F. palmata* Forsk, belong to the family Moraceae and were collected from herbal garden of Pharmacy Department, Integral University, India. Plant was authenticated by Dr. Tariq Husain, National Botanical Research Institute, Lucknow, India, and has been deposited in Herbarium with accession number 97960. Plants were shed and dried and powder (20 gram) was sequentially extracted with n-hexane (n-hex), dichloromethane (DCM), ethyl acetate (EtOAc), methanol (MeOH), and aqueous solvents in soxhlet apparatus until it turned colorless. The solvent was removed, filtered, and dried at room temperature and residues were stored at −20°C for future use. The percentage yield of different extracts of* F. palmata *were as follows:* F. palmata* leaves n-hex extract (FPLH) −3.05%,* F. palmata* leaves DCM extract (FPLD) −0.9%,* F. palmata* leaves EtOAc extract (FPLE) −1.25%,* F. palmata* leaves MeOH extract (FPLM) −9.15%,* F. palmata* leaves aqueous extract (FPLA) −3.85%,* F. palmata* bark n-hex extract (FPBH) −1.9%,* F. palmata* bark DCM extract (FPBD) −0.45%,* F. palmata* bark EtOAc extract (FPBE) −0.45%,* F. palmata* bark MeOH extract (FPBM) −4.9%, and* F. palmata* bark aqueous extract (FPBA) −4.95%.

### 2.2. Phytochemical Screening and Determination of Total Phenolic Content (TPC)

In order to identify the phytochemicals present in various extracts of* F. palmata*, qualitative chemical tests were carried out by using standard procedure [23]. Total phenolic content of the extracts was determined by using Folin-Ciocalteu's reagent (FCR) [24] and expressed as *μ*g gallic acid equivalent (GAE) in mg dry weight of extract.

### 2.3. *In Vitro* Antioxidant Assay

#### 2.3.1. DPPH Free Radical Scavenging Assay

The radical scavenging assay was measured according to the method of Brand-Williams et al. [25] by using DPPH as a free radical initiator model. Ascorbic acid was used as standard. The scavenging activity of DPPH radicals was measured by using the following equation:

(1) Percent  inhibition =[(control  absorbance−sample  absorbance)control  absorbance]×100.

Further, final result was expressed as IC_50_ value that represented the concentration of the extract producing 50% inhibition of DPPH radicals.

#### 2.3.2. Ferric Reducing Antioxidant Power Assay

Ferric reducing antioxidant power (FRAP) assay of different fractions of* F. palmata* extract was done by following the procedure of Benzie and Strain [26] with some modification [27]. Briefly, an aliquot (100 *μ*L) of an extract (with appropriate dilution) was added to 3 mL of FRAP reagent (10 parts of 300 mM sodium acetate buffer at pH 3.6, 1 part of 10 mM 2,4,6-Tri(2-pyridyl)-s-triazine solution, and 1 part of 20 mM FeCl_3_) and the reaction mixture was incubated in a water bath at 37°C. The increase in absorbance at 593 nm was measured after 30 min in Eppendorf spectrometer. The standard curve was plotted using ferrous sulphate solution, and results were expressed as *μ*M Fe (II)/mg dry weight of extract.

#### 2.3.3. Superoxide Radical Scavenging Activity

The super oxide radical scavenging assay was done according to the method of Kunchandy and Rao [28] with some modifications. Briefly, 10 *μ*L of nitro blue tetrazolium [1 mg/mL solution in dimethyl sulphoxide (DMSO)], 30 *μ*L of the extract or standard (ascorbic acid), and 100 *μ*L of alkaline DMSO (1 mL DMSO containing 5 mM sodium hydroxide in 0.1 mL water) were added to give a final volume of 140 *μ*L. The samples were incubated for 5 min at room temperature and the absorbance was measured at 560 nm using 96-well microtiter plate using microplate reader. The inhibition percentage was calculated as follows:

(2) Percent  inhibition=[(Absorbance  test  sample−Absorbance  control  sample)Absorbance  test  sample]×100.

#### 2.3.4. Assay for* In Vitro* Antilipoperoxidative Activity

Lipid peroxidation inhibition activity of* F. palmata* extracts was estimated as thiobarbituric acid reacting substances (TBARS) by the method of Ohkawa et al. [29] with some modification [30]. Quercetin was used as reference standard. The percent inhibition activity and IC_50_ value were calculated as described in Section 2.3.1.

#### 2.3.5. Assay for Oxidative DNA Strand Breaks

DNA scission induced by Fenton's reagent was demonstrated by using supercoiled pUC18 (2686 bp) plasmid DNA according to the method of Lee et al. [31] with slight modifications. Briefly, plant extracts (FPBA and FPLM) were used to protect the oxidative plasmid DNA (100 ng) damage which was initiated by Fenton's reagent [H_2_O_2_ (30 mM), ascorbic acid (100 *μ*M), and FeCl_3_ (160 *μ*M)], and the final volume of the mixture was raised up to 20 *μ*L. The mixture was then incubated for 45 min at 37°C and electrophoresis was carried out in Tris-acetate-EDTA buffer (40 mM Tris base, 16 mM acetic acid, and 1 mM EDTA, pH 8.0) for 1.5 h (40 V/20 mA). Mannitol was used as a positive control.

### 2.4. *In Vitro* Modulation of HMG-CoA Reductase Inhibitory Activity by Plant Extract

The HMG-CoA reductase assay kit from Sigma-Aldrich (St. Louis, MO, USA) with the catalytic domain of the human enzyme (recombinant GST fusion protein expressed in* E. coli*) was used, under conditions recommended by the manufacturer, to identify the most effective fraction of plant extract. The concentration of the purified human enzyme stock solution (Sigma) was 0.52–0.85 mg protein/mL. Reference statin drug pravastatin (from Sigma) was used as positive control. To characterize HMG-CoA reductase inhibition under defined assay conditions, reactions containing 4 *μ*L of NADPH (to obtain a final concentration of 400 *μ*M) and 12 *μ*L of HMG-CoA substrate (to obtain a final concentration of 400 *μ*M) in a final volume of 0.2 mL of 100 mM potassium phosphate buffer, pH 7.4 (containing 120 mM KCl, 1 mM EDTA, and 5 mM DTT), were initiated (time 0) by the addition of 2 *μ*L of the catalytic domain of human recombinant HMG-CoA reductase and incubated in Eppendorf BioSpectrometer (equipped with thermostatically controlled cell holder) at 37°C in the presence or absence (control) of 1 *μ*L aliquots of drugs dissolved in DMSO. The rates of NADPH consumed were monitored every 20 sec for up to 15 min by scanning spectrophotometrically.

IC_50_ value was calculated as described above and the % inhibitory enzymatic activity was calculated by using the following formula [17]:

(3) %Inhibition =[ΔAbsorbance  control−ΔAbsorbance  testΔAbsorbance  control]  ×100.

### 2.5. Liquid Chromatography-Mass Spectrometry (LC-MS) Analysis

The fraction which showed maximum inhibition of HMG-CoA reductase activity, that is, FPBA extract, was subjected to LC-MS analysis according to the protocol of Beer et al., [32] at advanced instrumentation research facility, Jawaharlal Nehru University, New Delhi, India. LC-MS and MS/MS analyses were conducted on a Waters Synapt G2 with 2D nanoACQUITY System equipped with a binary pump, in-line degasser, autosampler, and column oven. The system was coupled to a Synapt G2 Q-TOF system (Waters) equipped with an electrospray ionization (ESI) source. LC-MS and MS/MS spectra were compared to literature to tentatively identify peaks and bioactive compound.

### 2.6. Statistical Analysis

For the entire assays, samples were analyzed in triplicate and the results were expressed as mean ± S.D. IC_50_ value was calculated by Origin version 6.0 Professional software, and the results were evaluated by using one-way analysis of variance (ANOVA) and two-tailed Student's* t*-test. Statistical significance was expressed as *P < 0.05 and **P < 0.01.

## 3. Results and Discussion

### 3.1. Phytochemical Screening and Determination of Total Phenolic Content (TPC)

**Figure 1:**
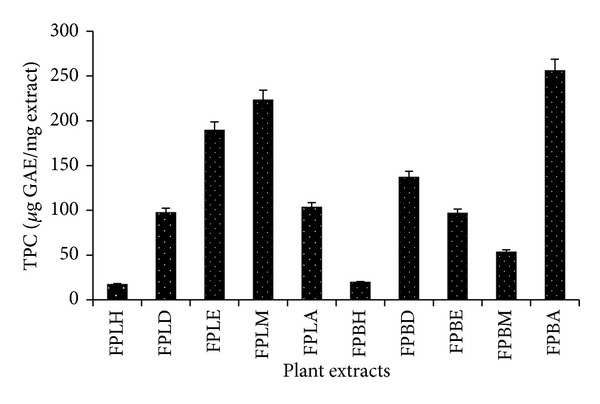
Total phenolic content of FPL and FPB extracts. Each value in the figure is represented as mean ± SD (*n* = 3).

The preliminary phytochemical screening of FPLM and FPBA extract showed the presence of tannins, terpenoids, saponins, phenols, carbohydrate, flavanoids, protein, glycosides, and reducing sugar, while FPLA and FPLE extract also revealed the above constituents except tannins and terpenoids (Table 1). In addition, the other extracts also include the varied amount of the above phytochemicals but to a lesser extent compared to FPLM and FPBA extract. Since, phenolic compounds are known as powerful chain-breaking antioxidants [33] and contribute directly to antioxidative action [34]; our results illustrate the TPC of various extracts of FPL and FPB extracts which were found to be in the following decreasing order: FPBA > FPLM > FPLE > FPBD > FPLA > FPLD > FPBE > FPBM > FPBH > FPLH. The result presented in Figure 1 clearly demonstrated that FPBA and FPLM extracts have better phenolic content (255.8 ± 2.72 and 223 ± 2.58 *μ*g GAE/mg of dry plant extract, resp.) than other extracts, whereas FPLH and FPBH have lowest phenolic content (17.2 ± 1.32 and 19.6 ± 1.58 *μ*g GAE/mg dry plant extract, resp.). Furthermore, the existence of considerable amount of TPC in FPBA is in fine agreement with prior phytochemical information on different parts of various* Ficus* species which have phenolic compounds as major components [35–38].

### 3.2. *In Vitro* Antioxidant Assay

**Figure 2:**
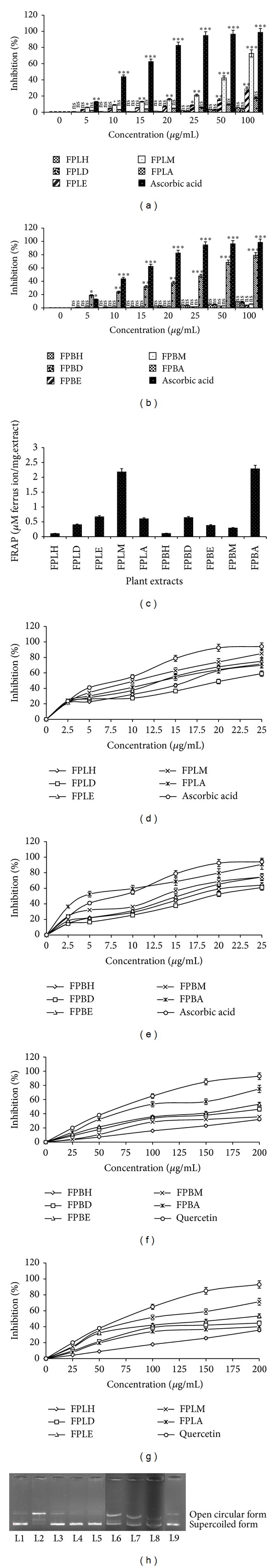
(a) DPPH free radical scavenging activity of different FPL extracts. Each value in the figure is represented as mean ± SD (*n* = 3). Nonsignificant (ns), significantly different *P < 0.05, **P < 0.01 versus 0 *μ*g/mL. (b) DPPH free radical scavenging activity of different FPB extracts. Each value in the figure is represented as mean ± SD (*n* = 3). Nonsignificant (ns), significantly different *P < 0.05, **P < 0.01 versus 0 *μ*g/mL. (c) Ferric reducing antioxidant power of different plant extracts. Each value in the figure is represented as mean ± SD (*n* = 3). (d) Percent inhibition of superoxide free radicals by different FPL extracts. Each value in the figure is represented as mean ± SD (*n* = 3). FPLH, FPLD, and FPLE are significantly different; *P* < 0.05 compared to control (0 *μ*g/mL); FPLM, FPLA, and ascorbic acid are significantly different; *P* < 0.01 versus 0 *μ*g/mL. (e) Percent inhibition of superoxide free radicals by different FPB extracts. Each value in the figure is represented as mean ± SD (*n* = 3). FPBH, FPBD, and FPBE are significantly different; *P* < 0.05 compared to control (0 *μ*g/mL); FPBM, FPBA, and ascorbic acid are significantly different; *P* < 0.01 versus 0 *μ*g/mL. (f) Percent inhibition of lipid peroxidation by FPL extracts. Each value in the figure is represented as mean ± SD (*n* = 3). FPLM, FPLA, and quercetin are significantly different; *P* < 0.05 compared to control (0 *μ*g/mL); FPLH, FPLD, and FPLE are significantly different; *P* < 0.01 versus 0 *μ*g/mL. (g) Percent inhibition of lipid peroxidation by FPB extracts. Each value in the figure is represented as mean ± SD (*n* = 3). FPBH and FPBE are significantly different; *P* < 0.05 compared to control (0 *μ*g/mL); FPBM, FPBA, FPBD, and quercetin are significantly different; *P* < 0.01 versus 0 *μ*g/mL. (h) Effects of various* Ficus virens* extracts on supercoiled pUC18 plasmid DNA (100 ng) damage caused by OH^•^ radicals. L1: pUC18 DNA + PBS (20 *μ*L), L2: pUC18 DNA + Fenton's reagent (10 *μ*L) + PBS (10 *μ*L), Lanes 3–5: pUC18 DNA + Fenton's reagent (10 *μ*L) + FPBA (50, 100, 250 *μ*g/10 *μ*L), Lanes 6–8: pUC18 DNA + Fenton's reagent (10 *μ*L) + FPLM (50, 100, 250 *μ*g/10 *μ*L), and Lane 9: pUC18 DNA + Fenton's reagent (10 *μ*L) + mannitol (250 *μ*g/10 *μ*L).

It is well known that antioxidants slow down or stop the oxidation of oxidizable material, by scavenging free radical and diminishing oxidative stress (excessive generation of ROS and RNS), which play a major role in the oxidation of varieties of biomacromolecules such as enzymes, proteins, DNA, and lipids that could result in the development of various diseases including atherosclerosis. Antioxidant activity of plant extract is associated with the presence of phenolic compound [39]; it is valuable to estimate the role of these sequentially extracted fractions in scavenging free radicals and determining the total antioxidant capacity. Our results showed that FPBA and FPLM extract exhibited significantly higher radical scavenging activity (IC_50_ = 23 ± 1.65 and 11.5 ± 0.51 *μ*g/mL, resp.) than other FPB and FPL extracts (Figures 2(a) and 2(b)). From the results presented in Figure 2, it is evident that there is a concentration dependent increase in radical scavenging activity of extracts, with substantial increase in FPBA extract,which is in agreement with previous reported data on* F. palmate,* which showed antioxidant activity and also observed no cytotoxicity to the normal peripheral blood mononuclear cells [40].

Moreover, the ability of plant extract to reduce ferric ions to ferrous ions is generally used as an index to determine the antioxidant power of extract [41]. The decrease in the concentration of ferric ion is a measure of the antioxidant activity of FPL and FPB extracts. As shown in Figure 2(c), FPBA extract possesses significantly higher antioxidant capacity (2.29 *μ*M Fe^2+^/mg) followed by FPLM (2.184 *μ*M Fe^2+^/mg) extract, while other extracts have not shown any significant activity. The ability of FPL and FPB extracts to scavenge superoxide radical, which is known to be a precursor of the more reactive oxygen species and results in various diseases, was also evaluated. Our data exemplified that superoxide radical scavenging capacity (IC_50_) of different extracts ranged from 3 ± 0.21 *μ*g/mL to 20 ± 1.15 *μ*g/mL (Figures 2(d) and 2(e)). Furthermore, FPBA extract exhibited significantly higher superoxide radical scavenging capacity (IC_50_ = 3 ± 0.21 *μ*g/mL), which was more than the standard ascorbic acid (IC_50_ = 10.8 ± 0.64 *μ*g/mL), followed by FPLM (IC_50_ = 11.2 ± 0.55 *μ*g/mL). In fact, several scientific studies reported that certain phytochemicals, such as flavonoids, ascorbic acid, tocopherol, tocotrienols, and polyphenols, have been reported as promising antioxidant compounds that might help in attenuating oxidative stress [11–13].

In order to evaluate the role of these extracts against various diseases, including atherosclerosis, which are mediated through excessive generation of free radicals, specifically by damaging DNA and lipid, inhibition of lipid peroxidation and oxidative DNA damage protective activity were evaluated. As revealed from Figures 2(f) and 2(g), all the extracts inhibited lipid peroxidation activity (IC_50_ = 88 ± 2.3 *μ*g/mL to >200 ± 4.25 *μ*g/mL), while significantly higher inhibition was observed by FPBA (IC_50_ = 88 ± 2.3 *μ*g/mL) and FPLM (IC_50_ = 94 ± 4.3 *μ*g/mL) extract when compared to standard quercetin (IC_50_ = 72 ± 1.6 *μ*g/mL). On the basis of the above results, it has been concluded that FPBA and FPLM possess significant antioxidant activity among all the tested extracts and were used for oxidative DNA damage protective activity.

Since hydroxyl radicals are involved in damaging DNA molecules, it is important to evaluate the protective role of these extracts at molecular level. The oxidative DNA damage protective activity of FPBA and FPLM extracts showed almost complete and partial protection against OH^•^-induced oxidatively damaged pUC18 plasmid DNA (Figure 2(h)). Hydroxyl radical generated during incubation of pUC18 plasmid DNA with Fenton's reagent indicates that OH^•^ generated from iron-mediated decomposition of H_2_O_2_ produced both single-strand and double-strand DNA breaks, which in turn was significantly attenuated by the addition of FPBA and FPLM extracts at different concentrations. It is well known that OH^•^ induced the oxidative damage in biological system and this type of damage, which is basically generated by the reaction between H_2_O_2_ and O_2_ in the presence of redox active metal, can be significantly reduced in the presence of potent antioxidant like plant extract and mannitol. The combined antioxidant results demonstrated that FPBA extract was the most potent free radical scavenger and also completely attenuates the oxidative DNA damage. This profound antioxidant property might be well correlated with high amount of phenolic content present in FPBA fraction (Table 3).

### 3.3. *In Vitro* Anti-HMG-CoA Reductase Activity and LC-MS Analysis

In addition to the physiological regulation of HMG-COA reductase, the human enzyme has been targeted successfully by drugs in the clinical treatment of high serum cholesterol levels and atherosclerosis [42]. The present work is a part of our drug discovery program, which mainly focuses on the development of new natural therapeutic agent with multiple targets and no toxicity after long term consumption. Paravastatin is the representative of the statin class of drugs that in their active hydrolysed form are specific inhibitors of HMG-CoA reductase. Statins share an HMG-like moiety, which may be present in the form of an inactive lactone form that acts a prodrug, and in addition they also have a rigid hydrophobic substituted decalin ring covalently bound to the HMG-like moiety. Thus, a dose response* in vitro* study was done to assess the inhibition of HMG-CoA reductase by sequentially extracted fractions of* F. palmata*.

**Figure 3:**
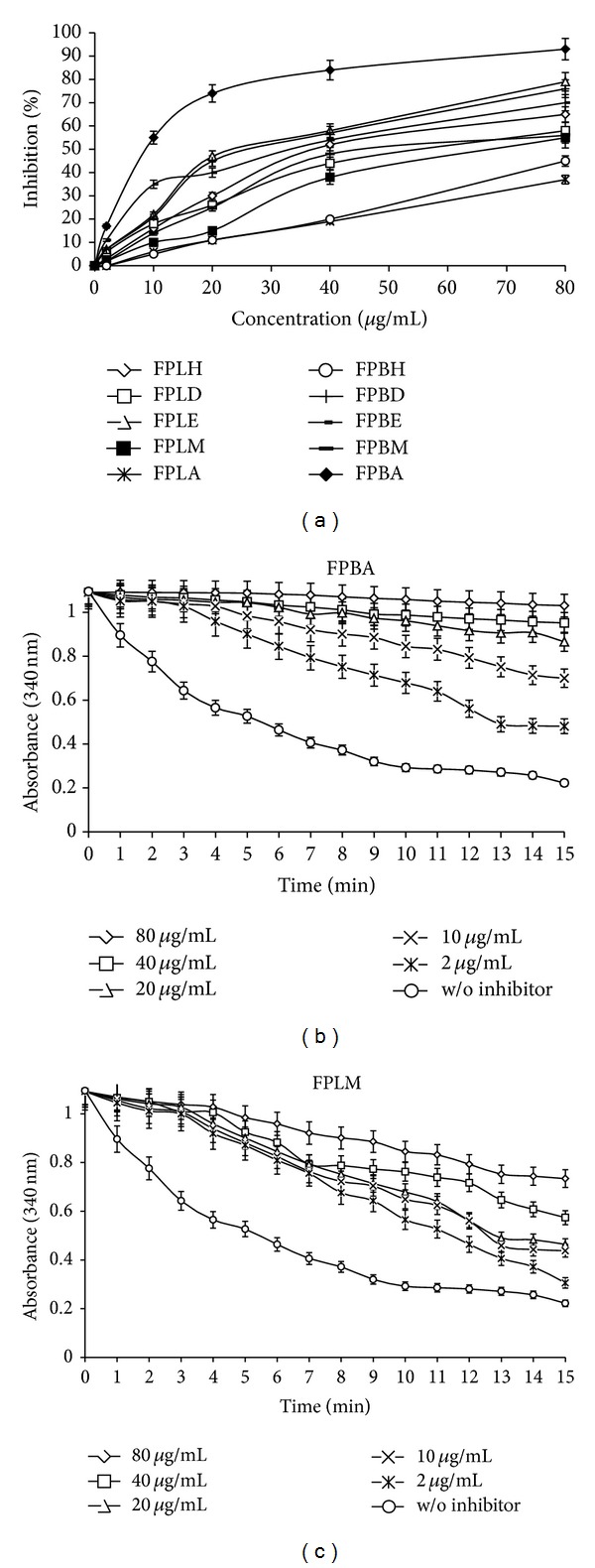
*In vitro* HMG-CoA reductase inhibitory activity of FPL and FPB extracts. Each value in the figure is represented as mean ± SD (*n* = 3). FPLH, FPLD, FPBE, FPBA and FPBH are significantly different; *P* < 0.05 compared to control (0 µg/mL); FPBM, FPLM, FPLA, FPBD, and FPLE are significantly different; *P* < 0.01 versus 0 µg/mL

In this context, our results initially demonstrated the capability of FPL and FPB extract to inhibit HMG-CoA reductase enzymatic activity in a cell-free assay system. For the standardization of the protocol, preliminary screening of pravastatin was done against HMG-COA reductase activity and observed 50% inhibition at 70.25 nM, which was in concordance with other studies [43]. From the data presented in Table 2 and Figure 3(a), it is evident that FPBA (IC_50_ = 9.1 ± 0.61 *μ*g/mL), FPLE (IC_50_ = 27 ± 1.21 *μ*g/mL), FPBD (IC_50_ = 30 ± 1.51 *μ*g/mL), FPBE (IC_50_ = 35 ± 1.84 *μ*g/mL), FPLH (IC_50_ = 38 ± 1.86 *μ*g/mL), FPBM (IC_50_ = 45 ± 2.13 *μ*g/mL), FPLD (IC_50_ = 58 ± 3.21 *μ*g/mL), and FPLM (IC_50_ = 65 ± 3.42 *μ*g/mL) extracts exhibited significant inhibition of HMG-CoA reductase activity, while other extracts were found to be nonsignificant. Our data clearly demonstrated that FPBA extract significantly inhibited HMG-CoA reductase activity with lowest IC_50_ value and also showed concentration dependent increase in inhibiting the enzymatic activity of HMG-CoA reductase. The spectrophotometric time scans demonstrated the ability of FPBA and FPLM at different concentrations (2–80 *μ*g/mL) to increasingly mimic the inhibitory activity which may suggest direct interaction of plant extracts with this enzyme (Figures 3(b) and 3(c)).

**Figure 4:**
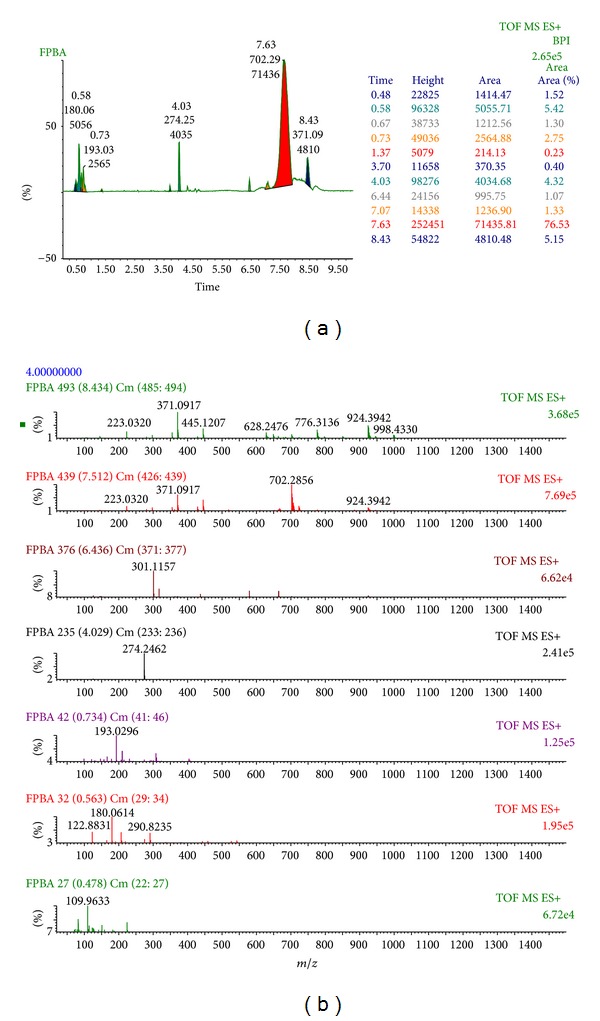
((a) and (b)) LC-MS/MS analysis of FPBA extract.

Further, FPBA fraction was subjected to LC-MS and MS/MS analysis, and spectra were compared to literature to tentatively identify peaks and bioactive compound (Figures 4(a) and 4(b)). LC-MS showed that extract does contain five prominent peaks having molecular weights 180.06, 193.03, 274.25, 702.29, and 371.09 au. Apart from these peaks there are several small peaks which may have also contributed to its enhanced activity. Alqasoumi et al. [44] claimed that aerial part of* F. palmata* was having five major constituents, including germanical acetate (*m/z*-491), psoralene (*m/z*-168), bergapten (5-methoxypsoralen,* m/z*-216), vanillic acid (*m/z*-168), and psoralenoside (*m/z*-368), which are responsible for its antioxidant and other medicinal properties, whereas in present study distinct peaks of molecular weights (*m/z*-180.06 and 371.09 au) were very similar to psoralene and psoralenoside. These chemical constituents might be responsible for inhibiting the catalytic activity of purified human HMG-CoA reductase in a cell-free assay through FPBA extract, and this extract might signify a novel class of HMG-CoA reductase inhibitors that can directly interact with this enzyme to obstruct the mevalonate pathway and prevent hypercholesterolemia. Further purification of this fraction is required to know the exact bioactive compound. From the above results, it was concluded that FPBA not only showed marked antioxidant activity but also exhibited significant HMG-COA reductase inhibitory property.

## 4. Conclusion

Based on our results, it has been concluded that FPBA extract not only possesses significant antioxidant and genoprotective properties but also is able to ameliorate the enzymatic activity of HMG-CoA reductase, which might suggest its role in combating hypercholesterolemia as well as in various oxidative stress-related diseases including atherosclerosis. Moreover, the LC-MS and MS/MS analysis of FPBA extract revealed five prominent peaks having molecular weights 180.06, 193.03, 274.25, 702.29, and 371.09 au. Further, preclinical and clinical studies are needed to explore the role of this extract and their bioactive compound isolated thereof in the prevention and management of cholesterol-induced oxidative stress and hypercholesterolemia as well as atherosclerosis.

## Figures and Tables

**Table 1:** Phytochemical screening of FPL and FPB extracts.

Plant extracts	Flavonoid	Tannins	Terpenoids	Phenols	Proteins	Carbohydrate	Saponins	Reducing sugar	Glycosides
FPLH	−	+	+	−	−	−	−	−	−
FPLD	+	++	+	+	+	−	−	+	+
FPLE	+	−	−	++	+	+	+	+	+
FPLM	++	+	+	+++	+	+	+	++	+
FPLA	+	−	−	+	+	+	++	+	+
FPBH	+	−	+	−	+	−	−	−	−
FPBD	+	+	++	++	−	−	−	−	−
FPBE	−	−	++	+	+	−	−	−	−
FPBM	−	−	−	+	−	−	−	++	−
FPBA	++	+	+++	+++	+	++	++	++	++

**Table 2:** IC_50_ value of different extracts of FPL and FPB against HMG-CoA reductase enzyme activity.

Plant extracts	IC_50_ value (µg/mL)
FPLH	38 ± 2.15*
FPLD	58 ± 3.15*
FPLE	27 ± 1.15**
FPLM	65 ± 3.24*
FPLA	NS
FPBH	NS
FPBD	30 ± 1.44**
FPBE	35 ± 1.64*
FPBM	45 ± 2.45*
FPBA	9.1 ± 0.53**
Pravastatin	70.25** nM

The values are expressed as mean ± SD (*n* = 3). Nonsignificant (NS), significantly different *P < 0.005, and **P < 0.001 versus without inhibitor.

**Table 3:** Correlation coefficient (*r* ^2^) values between various antioxidant methods and total phenolic content of FPBA extract.

Assay	Correlation coefficient (*r* ^2^)
TPC-DPPH	0.842
TPC-superoxide	0.988
TPC-lipid peroxidation	0.939
TPC-HMG-CoA reductase	0.803

## Abbreviations

FPL: *Ficus palmata* leaves
FPB: *Ficus palmata* bark
DPPH: 2, 2-Diphenyl-1-picrylhydrazyl
FRAP: Ferric reducing antioxidant power.
